# Time to diagnosis and treatment of pulmonary tuberculosis in indigenous peoples: a systematic review

**DOI:** 10.1186/s12879-023-08098-y

**Published:** 2023-03-07

**Authors:** Marie Varughese, Courtney Heffernan, Michael Y. Li, Richard Long

**Affiliations:** 1 Department of Mathematics and Statistical Sciences, School of Public Health, University of Alberta, 632 Central Academic Building, Edmonton, AB T6G2G1 Canada; 2 Faculty of Medicine and Dentistry, University of Alberta, Edmonton, AB T6G2R7 Canada; 3 Department of Mathematics and Statistical Sciences, University of Alberta, Edmonton, AB T6G2G1 Canada; 4 Faculty of Medicine and Dentistry, School of Public Health, University of Alberta, Edmonton, AB T6G2R7 Canada

**Keywords:** Tuberculosis transmission, Diagnosis delay, Patient and health system delay, Time to diagnosis, Time to treatment, Indigenous, Total delay

## Abstract

**Background:**

Time to diagnosis and treatment is a major factor in determining the likelihood of tuberculosis (TB) transmission and is an important area of intervention to reduce the reservoir of TB infection and prevent disease and mortality. Although Indigenous peoples experience an elevated incidence of TB, prior systematic reviews have not focused on this group. We summarize and report findings related to time to diagnosis and treatment of pulmonary TB (PTB) among Indigenous peoples, globally.

**Methods:**

A Systematic review was performed using Ovid and PubMed databases. Articles or abstracts estimating time to diagnosis, or treatment of PTB among Indigenous peoples were included with no restriction on sample size with publication dates restricted up to 2019. Studies that focused on outbreaks, solely extrapulmonary TB alone in non-Indigenous populations were excluded. Literature was assessed using the Hawker checklist. Registration Protocol (PROSPERO): CRD42018102463.

**Results:**

Twenty-four studies were selected after initial assessment of 2021 records. These included Indigenous groups from five of six geographical regions outlined by the World Health Organization (all except the European Region). The range of time to treatment (24–240 days), and patient delay (20 days–2.5 years) were highly variable across studies and, in at least 60% of the studies, longer in Indigenous compared to non-Indigenous peoples. Risk factors associated with longer patient delays included poor awareness of TB, type of health provider first seen, and self-treatment.

**Conclusion:**

Time to diagnosis and treatment estimates for Indigenous peoples are generally within previously reported ranges from other systematic reviews focusing on the general population. However among literature examined in this systematic review that stratified by Indigenous and non-Indigenous peoples, patient delay and time to treatment were longer compared to non-Indigenous populations in over half of the studies. Studies included were sparse and highlight an overall gap in literature important to interrupting transmission and preventing new TB cases among Indigenous peoples. Although, risk factors unique to Indigenous populations were not identified, further investigation is needed as social determinants of health among studies conducted in medium and high incidence countries may be shared across both population groups.

*Trial registration* N/a.

**Supplementary Information:**

The online version contains supplementary material available at 10.1186/s12879-023-08098-y.

## Introduction

Tuberculosis (TB) is one of the leading causes of death worldwide with 1.6 million dying from the disease in 2021, an increase from 1.4 million in 2019 [1]. In many parts of the world, the COVID-19 pandemic has resulted in a substantial reduction in TB testing and case notification, with an associated increase in mortality, taking TB control back by roughly 10 years [2]. The elimination of TB among individuals requires the early detection of active disease, reduction of the reservoir of TB infection, and a high rate of treatment completion [3, 4]. Risk factors associated with progression to active disease and/or TB transmission include chronic illnesses, low socioeconomic status, inequity, poor healthcare access, and a prolonged time to diagnosis and treatment [5]. At every step, the complex interplay between biological, social, environmental, and economic issues contributes to infection and disease [5].

Delayed diagnosis and treatment has been identified as a major contributor to TB transmission [4, 6]. Early detection of TB cases acquired from recent transmission represents a critical point of intervention to further reduce the reservoir of latent infection and prevent advanced disease, further transmission, and mortality [7–9]. These cases include those that exhibit symptoms related to disease such as cough or subclinical cases that are diagnosed through radiological or microbiological abnormalities either by health-seeking or active case finding. The interruption of TB transmission through early diagnosis and treatment is complicated by both patient and health system level factors. Previously conducted systematic reviews have reported total delay or time from onset of symptoms to treatment of pulmonary TB averaging between 25 and 185 days for both low, middle, and high-income countries [4, 10, 11]. Risk factors for increased time to treatment included poor access to healthcare, initial visits to traditional or private versus publicly funded practitioners, poverty, substance use, extrapulmonary TB, smear-negative TB, HIV, self-treatment, stigma, and a lack of knowledge about TB [4, 7, 12–15].

Systematic reviews about time to diagnosis and treatment have not focused on Indigenous peoples, who experience rates of TB that are generally higher than non-Indigenous persons [16]. Impacts of colonization through efforts of assimilation using residential schools, loss of lands, and historical trauma related to TB have contributed in different ways to the challenges facing access to care related to TB [17–20]. These colonial impacts along with evidence of on-going transmission in Indigenous populations could indicate longer delays in diagnosis and treatment [21]. A better understanding of the factors involved in diagnosis and treatment delay among Indigenous peoples is important to identifying gaps in knowledge and practice that could reduce the rate disparity. This systematic review assesses and summarizes literature about time to diagnosis and treatment of pulmonary TB (PTB) among Indigenous peoples worldwide. PTB was the main focus of this systematic review since it is the main disease site associated with transmission events. This assessment aims to include, where possible, the comparisons of diagnosis and treatment delay estimates between Indigenous peoples and non-Indigenous persons, to identify any additional risk factors unique to Indigenous groups.

## Methods

### Protocol and registration

Registration of the study protocol is on PROSPERO International prospective register of systematic reviews (PROSPERO August 2018: CRD42018102463). The study protocol is accessible at https://www.crd.york.ac.uk/PROSPERO/display_record.php?RecordID=102463. The systematic review followed the PRISMA framework [16, 22].

### Search strategy and selection criteria

The systematic review focuses on studies estimating the time, and associated risk factors (where available) for diagnosis and treatment delay among Indigenous peoples worldwide. Indigenous peoples include those who are either defined, or self-identified as belonging to this population group in each study [16, 23, 24]. Table 1 outlines terminology used to define diagnosis and treatment delays among studies assessed in the context of this systematic review.

**Table 1:** Glossary of definitions

Name	Definition
Indigenous peoples	Those who self-identified as belonging to this population group defined previously in literature [16, 23, 24]
Low incidence countries	Countries with TB incidence less than 15 cases per 100,000 population
Medium and/or high incidence countries	Countries with TB incidence of at least 15 cases per 100,000 population
Diagnosis delay (DD) /Time to diagnosis (TDx)	The duration between the onset of symptoms and diagnosis, the latter defined as a positive acid-fast bacilli (AFB) smear and/or nucleic acid amplification test, positive culture of a *Mycobacterium tuberculosis* complex organism or clinical diagnosis
Total delay (TD) /Time to treatment (TTm)	Duration between the onset of symptoms and treatment, which is commonly stratified into two main parts, patient delay and health system delay
Health System Diagnostic delay (DCD)	Duration between the first visit with a medical professional to diagnosis as defined above
Health System Treatment delay (TmD)	Duration between diagnosis (as defined above) and treatment
Patient delay (PD)	The time from onset of symptoms to first visit with a medical professional
Health system delay (HD)	The time from the first visit with a medical professional to start of treatment
Cut-off threshold	Either defined as a median estimate or arbitrarily chosen within a study to differentiate between a delayed or timely event
Delayed event	Any duration greater than the cut-off threshold
Timely event	Any duration less than or equal to the cut-off threshold

Herein we report synonymous terms, diagnosis delay (DD) or time to diagnosis (TDx) as the duration between the onset of symptoms and diagnosis (see Table 1). Time to treatment (TTm) or total delay (TD) is reported as the duration between the onset of symptoms and treatment, which is commonly stratified into two main parts, patient delay (PD) and health system delay (HD). The threshold used to define the cut-off of a “delayed event” is generally reported as a median estimate within a study, though sometimes it may be an arbitrarily chosen point-estimate. As such, a “delayed event” is any that occurs at a time after the cut-off threshold (median or otherwise), and a “timely event” is any that occurs in the time before the cut-off threshold.

The review includes manuscripts in peer-reviewed journals or published conference abstracts between 1910 and 2019 (see below) since the search was conducted mid-year in 2020 and to help minimize impacts on time to diagnosis and/or treatment estimates for TB during the COVID-19 pandemic. There were no restrictions on language and sample size. Articles in non-English languages were translated to verify that the inclusion criteria were satisfied. Literature that included Indigenous and non-Indigenous populations were accepted as part of the review if time to diagnosis and/or treatment results were stratified by population group. Studies with diagnosis and/or treatment delay estimates that were based on pulmonary TB in combination with extrapulmonary TB cases were included. These studies were either annotated in result tables as including ‘pulmonary and extrapulmonary TB’ or where possible, the PTB estimate was provided. Risk factors associated with delay in diagnosis and/or treatment were assessed as a secondary focus.

Studies that reported time to diagnosis and/or treatment estimates in only extrapulmonary TB cases or included only non-Indigenous populations were excluded. Definitions that did not include start and end points as part of the definition for time to diagnosis and/or treatment terminology (see Table 1) were excluded. Outbreak studies were excluded as they report estimates for time to diagnosis for an index case, rather than providing population level estimates. The focus of this review is PTB, given its importance to transmission.

Data (literature) was collected from Ovid and PubMed in June 2020. Ovid databases accessible to the University of Alberta included Embase (1974–June 12, 2020), Global Health (1910–June 12, 2020), Ovid Medline (1946–June 12, 2020), and Journals@Ovid Full Text (up to June 12, 2020). The PubMed search was conducted June 15, 2020. The literature search included broad and narrow search terminology about TB, Indigenous peoples or groups, and time to diagnosis and treatment terminology. Table 2 describes the search terms, which are grouped into three themes: (1) TB, (2) time to diagnosis or treatment, and (3) Indigenous peoples. The terms described in Table 2 were specific to the Ovid databases. Additional file 1: Appendix A1 describes analogous MESH terms used in PubMed. The search terms for Indigenous peoples were obtained from a systematic review conducted by Tollefson et al. that quantified the burden of TB disease among Indigenous peoples [16]. The review by Tollefson et al. in turn obtained Indigenous group names from Bartlett et al. and organizations participating in the United Nations Permanent Forum on Indigenous Issues (UNPFII) [16, 24].

**Table 2:** Keywords used for search strategy in the systematic review of delay in diagnosis and treatment studies among Indigenous peoples for Ovid databases

Theme	Search terms*
Tuberculosis	TB OR tuberculosis OR pulmonary TB OR pulmonary tuberculosis
Time to Diagnosis/Treatment OR Diagnosis Delay/Total delay^†^	diagnos* delay OR treatment delay OR treatment seek* OR time delay OR delay* in diagnos* OR delay* in treatment OR “time to diagnosis” OR “time to treat*” OR total delay OR patient delay OR health system* delay OR health provider* delay OR health seek* period OR doctor delay
Indigenous Peoples^‡^	american native continental ancestry group/ OR indigenous OR "indigenous people*" OR "indigenous population*" OR first nation* OR inuit OR metis OR aborigin* OR torres strait islander* OR maori OR cook islander* OR tribe OR tribal OR eskimo* OR north american/ OR inuits/ OR oceanic ancestry group/ OR american indian OR native american OR alaska natives OR roma OR bushmen OR herdsmen OR hill people OR amazon* OR lahu OR akha OR mon OR lua OR mbri OR karen OR hmong OR miao OR hui OR (minority AND china) OR aka OR babenjelle OR babongo OR bacwa OR bagyeli OR baka OR bakola OR bakoya OR bambuti OR batwa OR pygmy OR aasax OR akie OR aweer OR barabaig OR dahalo OR datoga OR elmolo OR hadzabe OR hadza OR maasai OR ogiek OR sandawe OR sengwer OR waata OR yaaku OR amazigh OR imazighn OR berbers OR tuareg OR afar OR aka OR babendjelle OR boranna OR dinka OR fulani OR kanuri OR karamajong OR manjo OR nuer OR peul OR pygymy OR tuareg OR tubu OR wodaabe OR bassari OR bororo OR daza OR nemadi OR ogoni OR teda OR khoekhoe OR khoikhoi OR basarwa OR khwe OR nama OR (san AND africa) OR tsumkwe OR aleut OR alutor OR chelkancy OR chukchi OR chulymcy OR chuvancy OR dolgan OR ency OR evenk OR itelmen OR kamchadal OR kereki OR kety OR khanty OR koryak OR kumandincy OR mansi OR nanaicy OR negidalcy OR nenets OR nganasan OR nivkhy OR orochi OR oroki OR saami OR sami OR selkup OR shorcy OR soioty OR tazy OR telengity OR teleuty OR tofolar OR tubolar OR tuvin-todjin OR udege OR ukagiry OR ulchi OR veps OR cherokee OR navajo OR choctaw OR sioux OR chippewa OR greenlandic inuit OR siberian inuit OR wa's nyoongar OR wongi OR tamitji OR koori OR tangata whenua OR Adivasi OR himba OR hausa OR yoruba OR igbo OR pastoralist* OR panara OR surui OR xavante OR arawak OR tukano OR maku OR arapaso OR baniwa OR desana OR kubeo OR hupda OR piratapuya OR tuyuca OR wanana OR tariana OR tukano OR amazonic OR ashanninka OR shipibo OR matsiguenga OR aguaruna OR shawi OR huambisa OR yanesha OR quichua-amazonian OR achuar OR cashibo-cacataybo OR nomastshiguenga OR quechua- lamistas OR amarakaire OR harakmbut OR shapra OR aymara-andes OR quechua-andes OR warao OR ache natives OR icana-aiari OR waupes-papuri OR tiquie OR yanomami OR "rio negro" OR anu OR bari OR wayuu OR yukpa OR yanomami OR "trio indians" OR bedouins OR chukotka autonomous indigenous OR "jawadhu-hills" OR saharia OR "scheduled tribes" OR "forest people*" OR "dongria kondh" OR "kutia kondh" OR "langia saora" OR "pandi bhuiyan" OR nicobarese OR gond OR "gond gawari" OR mana OR pawara OR "raj gond" OR bharia OR bhil OR baiga OR "pacific islanders" OR "e-lun chun" OR "papua new guineans" OR bai OR chaoxian OR kazakh OR li OR manchu OR mongols OR menggu OR tujia OR uyghur OR zhuang OR dogon OR "brazillian native indian" OR chine OR "zulu state indigenous" OR "madhya pradesh tribal" OR "native hawaiians"

*Search Terms for Tuberculosis AND Time to diagnosis or treatment AND Indigenous populations

^†^“Time to diagnosis”/“Time to treatment” used instead of “Time to diagnos*“/”Time to treat*” for the PubMed search

^‡^Narrow key word search terminology obtained from Tollefson et al. and Bartlett et al. [16, 24]

M. Varughese conducted the search, literature extraction, and duplicate record removal. M. Varughese and M. Li independently screened titles and abstracts for those that warranted a full-text review. M. Varughese and C. Heffernan subsequently assessed full-texts for inclusion. Disagreements at the first and second screening stages were resolved through consultation with C. Heffernan and M. Li, respectively. Articles written in non-English language were translated and/or assessed for inclusion.

### Data synthesis

Data extracted from each selected paper included the year of study, country, Indigenous population group, total sample size (and stratified sub-population size for studies that included Indigenous peoples), study design/sampling description, study’s primary objective, study setting, type of data (disaggregate/single population study), definition of the time to diagnosis and treatment terminology, estimate (e.g. mean/median), estimate type (descriptive/univariate, multivariate, and/or qualitative), and risk factors associated with longer time to diagnosis and/or treatment if available. Disaggregated studies were defined as those reporting Indigenous population group estimates independently.

For estimates, preference where possible was given to median and/or mean values. Otherwise, percentiles (rank-based estimates) were used to describe the relative position compared to the median value. For example, if a study stated that 30% of people had a delay greater than 45 days, this estimate would be analogous to a 70th percentile or “above median”. This would mean the 50th percentile/median value would be less than 45 days. If a study stated that 80% of people had a delay greater than 45 days, this estimate would be analogous to a 20th percentile or “below median”. This would indicate that the 50th percentile/median value is greater than 45 days. The use of “below median” and “above median” provide context of cut-off values and comparisons to other studies. Estimates provided as a range were included if mean, median, and/or percentile values were not available. Estimations of time to diagnosis and/or treatment across studies was preferentially reported using medians, means, 35th to 65th percentiles, and mid-point of range values.

Risk factors were grouped into four main categories: demographics, language/education/culture, TB specific, and health care access. Demographics factors included age, gender, and indigenous status. Language/education/culture factors included language barrier, education status/TB knowledge, and stigma/cultural taboos. TB specific factors included AFB smear status/extrapulmonary TB and misdiagnosis. Health care access factors included type of medical location first visited, type of medical professional first visited, self-treatment/not seeking care, distance to health facility, transportation barriers, cost of treatment, and administrative delays. Risk factors when assessed were documented by type of delay, risk factor, and those that showed significance to a delayed or timely event. Method type was stratified as multivariate, descriptive/univariate, and/or qualitative. For multivariate and descriptive/univariate methods, factors that had a p-value less than 0.05 were described as significant. The significance level would be based on how authors chose to analyze the data i.e. selecting cut-off values of time to event estimates based on central tendency, percentiles, or a priori. Themes that arose from qualitative methods were noted as having significance to a delayed or timely event.

Selected literature was qualitatively assessed using the Hawker checklist for sampling, overall methodology, and generalizability of the study. This checklist has nine criteria each rated as very poor (point = 1), poor (point = 2), fair (point = 3), and good (point = 4) totaling 36 points [25]. The nine criteria are: (1) abstract and title, (2) introduction and aims, (3) method and data, (4) sampling, (5) data analysis, (6) ethics and bias, (7) results, (8) transferability or generalizability, and (9) implications and usefulness [25]. Two reviewers, M. Varughese and C. Heffernan assessed literature independently. The assessment scores of both reviewers were averaged where possible or deferred to a lower score to provide an overall conservative assessment. Since the scoring scale is arbitrary, a score of 27 and above was considered a ‘good quality study’. This minimum value was obtained by considering an article scoring at least three (interpretation of fair) across the nine categories.

## Results

**Fig. 1:**
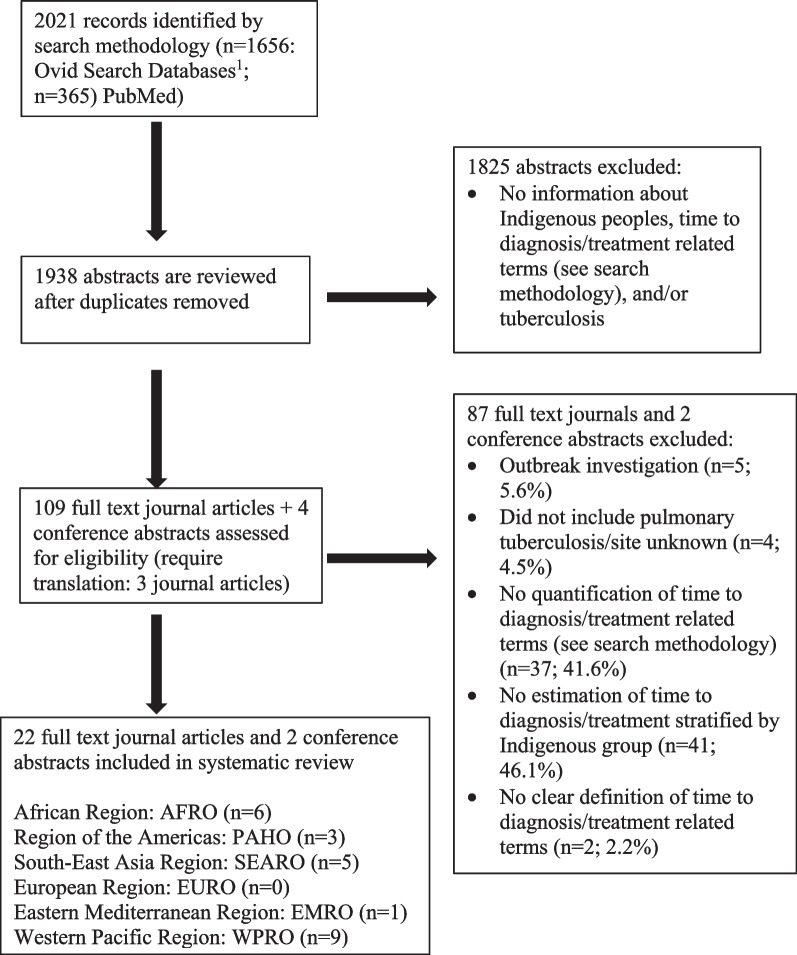
Search methodology results and selection of studies for the systematic review

Figure 1 describes the total studies obtained, screened, and selected for the systematic review. A total of 2021 records were obtained from Ovid search databases (n = 1656) and PubMed (n = 365). Removing duplicate records yielded a total of 1938 records. A total of 1825 records were excluded due to lack of information about time to diagnosis or treatment of TB among Indigenous peoples. One-hundred and thirteen records (109 articles and four conference abstracts including three requiring translation) assessed for eligibility resulted in 24 records (22 articles and two conference abstracts) included in the systematic review (see Fig. 1). A total of 89 articles (87 full-text articles and two conference abstracts) were excluded due to outbreak investigations (n = 5; 5.6%), no inclusion of pulmonary tuberculosis (n = 4; 4.5%), no clear definition of time to diagnosis/treatment related terms (n = 2; 2.2%), no quantification of time to diagnosis/treatment related terms (n = 37; 41.6%) or estimates stratified by Indigenous group (n = 41; 46.1%). One article excluded due to no estimation of delay estimates by Indigenous group could not be further assessed beyond the abstract due to full-text inaccessibility [26]. Selected studies represented five out of six World Health Organization regions: African Region (n = 6), Region of the Americas (n = 3), South-East Asia Region (n = 5), Eastern Mediterranean Region (n = 1), and Western Pacific Region (n = 9).

Table 3 provides a summary of selected studies such as region, population group, sample size, primary focus, study period and design, sampling method, type of data (single/disaggregate), and delay estimates. Time to diagnosis and/or treatment estimates were reported as means, median, percentiles, and/or range values. The primary focus in six of 24 studies (25%) was not related to estimating time to diagnosis and/or treatment [27–32]. Of these studies, four provided percentile/range estimates [28, 29, 31, 32]. Across all studies, 50% (12/24) provided percentile/range estimates for time to diagnosis and/or treatment.

**Table 3:** Summary of delay in diagnosis by study (n = 24) among indigenous populations

Year of Study	Country (Total Sample Size)/Indigenous Population Group (Total)	Study Design/Primary Objective was to estimate delay (Yes/No)	Study Setting/Sampling Method/Description	Type of Data (Disaggregate/Single)	Definition of Event	Estimate, (Type)	Study
2012–2014	Canada/Inuit (n = 344)	Prospective pragmatic/No	Nunavut/Xpert MTB/RIF tests in patients with suspected TB	Single	TmD: Receipt of sputa by the laboratory to treatment (Tests: Xpert MTB/RIF & three smears)	GX (Smear+/Smear−): 1.8 days (Mean) Smear+: 7.7 days (Mean) Smear− (Culture): 37.1 days (Mean)	[27]
2002–2006	Taiwan (n = 78,118)/Aboriginal (n = 2,530)	Cross-sectional/Yes	Taiwan/Surveillance data	Disaggregate	DCD: First medical examination to diagnosis (positive smear /culture/clinical symptoms) TmD: Diagnosis to treatment initiation	DCD: 9 ± 23 days (Mean) TmD: 9 ± 38 days (Mean)	[33]
1992–2001	New Zealand (n = 244)^†^/Maori (n = 110) & Pacific Islanders (n = 4)	Descriptive (Quantitative)/No	The Waikato Health District/Surveillance data	Disaggregate	TDx: Onset of symptom to case notification: diagnosis date or treatment date/clinical signs (if bacteriological & histological evidence is absent)	28 days (15th percentile)^‡^	[28]
2009–2010	Ethiopia (n = 216)^†^/Pastoralists (n = 91)	Cross-sectional/Yes	Afar Region/Sampling of TB patients in two health facilities	Disaggregate	PD: Onset of symptom to first visit to health provider HD: Time between first visit to health provider and start of treatment TTm/TD: Onset of symptom to TB treatment	PD: 20 days (40th percentile) HD: 33.5 days (48th percentile) TD: 70.5 days (44th percentile)	[3]
2007	India/Tribal (n = 261)^§^	Cross-sectional/Yes	Mayurbhanj district, Odisha/Sampling of TB patients in four health units	Single	PD: Onset of symptom to first health facility visit HD: First health facility visit to start of TB treatment TTm/TD: Onset of symptom to start of TB treatment	PD: 24 days (Median)/36.5 days (Mean) HD: 3 days. (Median)/11.6 days (Mean) TD: 24 days (Median)/37.5 days (Mean)	[37]
2011–2012	Solomon Islands/Kwaio people (n = 16: n = 4, 1 focus group n = 12)^†^ Note: Delay estimate based on n = 3	Descriptive (Qualitative)/Yes	East Kwaio/Hospital records and focus groups	Single	PD: Duration between feeling unwell and presenting to hospital for diagnosis and treatment	2–3 years (range)	[32]
1998–2000	Thailand (n = 557)^§^/Hill Tribe (n = 74)	Cross-sectional/Yes	Chiang Rai/Sampling of TB patients in one hospital	Disaggregate	PD: Onset of cough (or other symptom if cough not present) to first visit with doctor/health staff DCD: First visit with doctor/health staff to TB diagnosis	PD: 21 days (49th percentile) DCD: 7 days (53rd percentile)	[39]
1985–1998	Australia (N = 375)^§^/Aboriginal & Torres Strait Islanders (n = 47)	Cross-sectional/Yes	Queensland/Surveillance data	Disaggregate	PD: Onset of symptom to first visit to a medical practitioner HD: First visit to a medical practitioner to start of TB treatment	PD: 30 days (Median) HD: 10 days (Median)	[40]
2000–2005	United States/Marshall Islanders (n = 40)^†^	Cross-sectional/No [Abstract]	Arkansas/Surveillance data	Single	TDx: Onset of symptom to TB diagnosis	60 days (35th percentile)	[29, 47]
2006–2009	United States/American Indian and/or Alaska Native (n = 111)^†^	Validation study/No	United States/Surveillance data and chart review	Single	TmD: Diagnosis (ICD-9 diagnostic codes for active TB) to TB treatment	0 days (Median) (13.9% > 7 days)	[30]
2004	Ethiopia/(n = 243)	Cross-sectional/Yes	SNNPR/Samp-ling of TB patients in one health centre	Single	PD: Onset of symptom to first consultation (e.g. health centre)	4.3 weeks (Median)/9.8 weeks (Mean)	[48]
2010–2012	Vanuatu/Melanesian (n = 35)^†^	Descriptive (Qualitative/Quantitative)/Yes	Vanuatu/Samp- ling of TB patients in four study areas	Single	TDx: Onset of symptom to TB diagnosis (defined as visit to hospital)	8 weeks IQR: 20 weeks (Median)	[35]
2006–2013	China (n = 76,486)§/Hmong (n = 125) & Tujia (n = 211)	Cross-sectional/Yes	Yunnan/Surveillance data	Disaggregate	PD: Onset of symptom to first contact with a doctor in a CDC TB center (DOTS facility)	90 days (63rd percentile)	[50]
2011–2013	India/Tribal (n = 580)^§^	Cross-sectional/Yes [Abstract]	Rayagada district, Odisha/Samp- ling of TB patients in 20 microscopy centres	Single	PD: Onset of symptom to first visit at a Designated Microscopy Centre	2 months (87th percentile)/1 month (63rd percentile)	[49]
2011	Ethiopia/Pastoralist (n = 129)	Cross-sectional/Yes	Bale Zone/Sampling of TB patients in four health facilities	Single	PD: Onset of symptoms to first visit at a health care facility HD: First health facility visit to start of TB treatment TTm/TD: Onset of symptoms to start of TB treatment	PD: 63 days (Median) HD: 34 days (Median) TTm/TD: 97 days (Median)	[41]
2007	Ethiopia/Pastoralist (n = 226)^†^	Cross-sectional/Yes	Jigjiga & Shinile Zones/Sampling of TB patients in TB management units from two zones	Single	PD: Onset of symptoms to first visit with a professional health provider DCD: First visit to a professional health provider to diagnosis TDx: Onset of symptoms to TB diagnosis	PD: 60 days (Median)/130 days (Mean) DCD: 6 days (Median)/9 days (Mean) TDx: 70 days (Median)/140 days (Mean)	[42]
2012–2013	Iran/Nomadic Tribes (Qashqai) (n = 4)	Community-based/No	Fars Province/Active surveillance of nomadic populations	Single	TDx: Onset of symptoms to TB diagnosis	6 weeks–52 weeks (Range)	[31]
2005–2006	Malaysia (n = 272)/Kadazan-Dusun-Murut, Bajau, Rungus and Brunei (n = 249)^§^	Cross-sectional/Yes	Sabah/Sampling of TB patients in 36 health centers	Disaggregate	PD: Onset of symptoms to first visit with a health provider (government and/or private)	30 days (48th percentile)	[51]
2003–2004	Malaysia (n = 316)/Iban, Bidayuh, & Melanau (n = 185)^§^	Cross-sectional/Yes	Sarawak/Sampling of TB patients in 10 TB clinics from three divisions	Disaggregate	PD: Onset of symptoms to first medical consultation DCD: First medical consultation to diagnosis	PD: 30 days (55th percentile) DCD: 22 days (56th percentile)	[38]
2001	India/Tribal (n = 37)†	Descriptive (Qualitative and Quantitative)/No	Andhra Pradesh/Snowball sampling of TB patients in four health centres from two mandals	Single	PD: onset of symptoms to first visit with a health provider (includes private, qualified/unqualified) TTm/TD: Onset of symptoms to start of TB treatment	PD: 4–7 months (range) TTm/TD: ≥ 8 months (max of 15 months)	[32]
2017–2018	Ethiopia (n = 442)/Pastoralist (n = 206)	Matched case–control/Yes	Somali Region/Sampling of TB patients in five health facilities	Disaggregate	PD: onset of symptoms to first visit with a health provider (in health center, hospital, and/or private facility with TB services)	30 days (48th Percentile)	[54]
2002	Vietnam (n = 2093)/Ethnic Minority (n = 124)^§^	Cross-Sectional Study/Yes	Vietnam/Sampling of TB units in 70 districts	Disaggregate	TTm/TD: Onset of symptoms to start of TB treatment	5 weeks (Median)/8.4 weeks (Mean)	[52]
1995	West Africa (n = 100)/Akan tribe (n = 86)^§^	Cross-Sectional Study/Yes	Ghana/Sampling of TB patients from one hospital	Disaggregate	TTm/TD: Onset of symptoms to start of TB treatment	5 months (Median)	[34]
2010–2014	China (n = 1166)/Minority (n = 1061)	Cross-Sectional Study/Yes	Jinping County/Surveillance data	Disaggregate	PD: Onset of symptoms to first doctor’s visit	30 days (51th Percentile)	[53]

Time to diagnosis (TDx)/Treatment (TTm), Health system delay (HD), Health System-Treatment Delay (TmD) & Diagnostic Delay (DCD), & Total Delay (TD)

^†^Include pulmonary and extrapulmonary TB

^‡^No significant association between time to diagnosis/treatment event by population group

^§^Include smear-positive pulmonary TB cases only

The range of time to diagnosis (56–203 days; 4 of 5 studies) and treatment (24–240 days; 6 studies), and patient (20 days–2.5 years; 15 studies), health system (3–34 days; 4 studies), diagnostic (6–22 days; 4 studies), and treatment (0–9 days; 3 studies) delay estimates were variable across studies. The time to diagnosis estimate from the Van der Oest et al. study was not included above since the percentile criteria (28 days—15th percentile) was not met. In the Banerjee et al. study, time to treatment was not provided as a range, but constructed using assumptions within the paper (Range: 8 to 15 months) [32]. Since the maximum value was not explicitly stated, the minimum value (8 months) was included in the above summary range for time to treatment.

Table 4 describes the qualitative assessment of studies (not including abstracts) using the Hawker’s checklist. The scores ranged between 21 and 33 out of a total score of 36. Qualitatively 77% (17/22) of studies had a score of at least 27, which was pre-defined as a ‘good quality study’. None received a score of one (‘very poor’) in the nine criteria. Ethics/bias (8/22; 36%) and transferability/generalizability (7/22; 32%) were two sub-categories that received the most ‘poor’ ratings (score = 2) across studies. Studies that received ‘poor’ ratings for ethics/bias did not state ethics [32–34] and/or limited their discussions of bias related to methods and data collected. Some studies used convenience or purposive samples [3, 32, 35, 36] and others required more sampling [28, 34, 37–39], which impacted the bias, transferability, and generalizability of results. Remoteness and heterogeneity of Indigenous communities within and across regions is an additional limitation to generalizing results from observational studies.

**Table 4:** Qualitative assessment of studies (n = 22) not including abstracts (n = 2) using the Hawker checklist

References^a^	Abstract & Title (/4)	Introduction and aims (/4)	Methods and data (/4)	Sampling (/4)	Data Analysis (/4)	Ethics and bias (/4)	Results (/4)	Transferability &generalizability (/4)	Implications & Usefulness (/4)	Total Score (/36)
Basa et al. [37]	4	3	3	3	3	2	4	2	3	25
Hussen et al. [41]	3	4	4	4	4	4	4	3	3	33
Lin et al. [33]	3	3	3	3	3	2	3	3	2	22
Gele et al. [42]	4	4	4	4	4	4	3	3	3	33
Ngamvithayapong et al. [39]	3	3	3	3	3	2	4	3	3	27
Alverez et al. [27]	3	3	3	3	3	3	4	3	4	29
Ward et al. [40]	4	3	4	4	4	3	4	3	3	32
Khan et al. [50]	2	4	4	4	4	3	4	3	3	31
Podewils et al. [30]	3	3	3	3	3	3	3	3	3	27
Cambanis et al. [48]	3	3	3	3	4	3	4	3	3	29
Belay et al. [3]	3	3	2	3	3	3	3	2	3	25
Oest et al. [28]	3	3	3	3	3	2	3	3	3	26
Viney et al. [35]	3	3	2	2	3	3	3	2	3	24
Honarvar et al. [31]	3	4	4	3	3	3	4	2	4	30
Massey et al. [36]	3	3	3	3	3	3	4	2	4	28
Rundi et al. [51]	3	3	4	3	4	3	4	3	3	30
Chang et al. [38]	4	3	3	2	2	2	3	2	3	24
Banerjee et al. [32]	3	3	2	2	2	2	3	2	2	21
Getnet et al. [54]	4	4	4	4	4	3	4	3	3	33
Huong et al. [52]	4	4	3	4	4	3	3	3	3	31
Lawn et al. [34]	3	4	4	4	3	2	4	3	4	31
Lingxing et al. [53]	4	4	3	3	3	2	4	4	4	31

^a^Abstracts were not assessed due to limited information: [29, 47, 49]

**Fig. 2:**
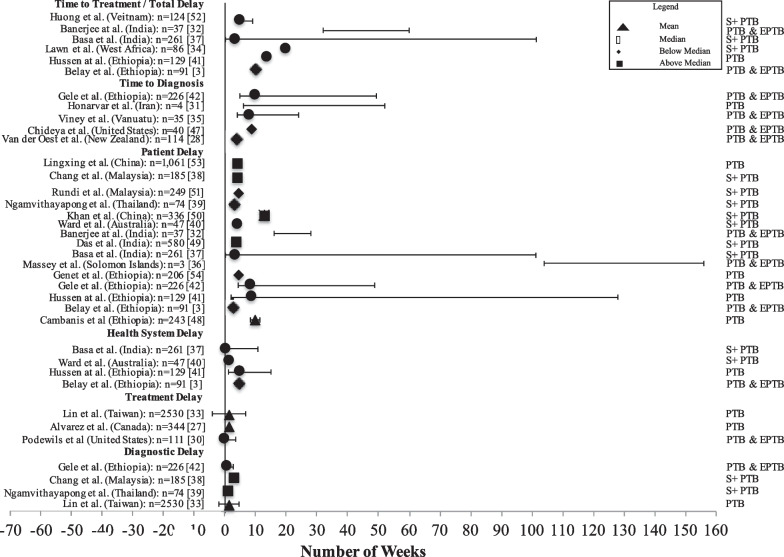
A forest plot that describes the distribution of estimate types related to time to diagnosis and/or treatment of TB among Indigenous peoples across literature included in the systematic review. a) Acronyms: pulmonary TB (PTB), smear positive pulmonary TB (S + PTB), and pulmonary and extrapulmonary TB (PTB & EPTB), b) Distribution was reconstructed to determine the interquartile range, median, and/or range values [30, 32, 35], c) Interval with no point estimate, represents a range [31, 32, 36], and d) Interquartile ranges were used to describe the spread for median values except in Basa et al. and Hussen et al. [37, 41], which was reported as a range

Figure 2 is a forest plot that describes the distribution of estimate types (time to diagnosis/treatment, patient/health system delay, and diagnostic/treatment delay) for each study included in the systematic review. Figure 2 includes other information such as sample size, uncertainty (mean: 95% confidence interval, median: interquartile (IQR), and range limits) where available, and TB site. Studies that reported other percentile quantities (also termed “below” or “above” median value) were included in Fig. 2. In these studies, uncertainty of estimates was not generally reported. One study [40] whose estimate was less than the 35th percentile was not included in Fig. 2.

Uncertainty of point estimates was either reported or reconstructed from data [35] within the study (if available) in 8 out of 23 studies (35%). Three studies reported a range without a point estimate [31, 32, 36]. Two studies reported range values (instead of IQR) with a median point estimate [37, 41]. There were no differences observed for treatment (n = 3 studies) and diagnostic (n = 4 studies) delay. Point estimates (mean, median, and 45th to 55th percentiles) for total delay had the largest variation ranging between 5 and 20 weeks. Point estimates for patient delay (3 to 10 weeks) contributed more to total delay compared to health system (< 1 week to 5 weeks) delay. Among studies that reported ranges, the greatest variability was observed in patient and total delay estimates. One study, conducted in the Solomon Islands, reported patient delays of between two and three years [36]. Seeking care from traditional healers, inaccessibility of healthcare due to remoteness, and cost were some of the factors explaining significantly longer patient delays compared to other studies.

Table 5 provides a summary of risk factors associated with time to event measures in 21 of 24 studies (88%). Two studies [27, 30] did not describe associated risk factors for time to event measures. Sixty-three percent of studies (15/24) described risk factors associated with patient delay. Risk factors were stratified into four themes: (1) demographics, (2) language, education, and culture, (3) TB specific, and (4) health care access and attributes such as type of study (Indigenous (S)/disaggregated population (D)) and methodology (multivariate/descriptive (e.g. chi-square test)/univariate/qualitative) were provided. For each risk factor, total references were estimated to examine overall strength in risk factor association with time to event measures. Multivariate, descriptive, and univariate methods were mainly used to assess risk factors associated with time to treatment/total delay and health systems delay (including treatment and diagnostic delay). More variability was observed in the methods (quantitative and qualitative) used to assess risk factors associated with time to diagnosis and patient delay.

**Table 5:** Risk factor summary across time to event measures, direction of association (delayed/timely), and type of study (Indigenous/disaggregate population)

Risk factor	Estimate Direction/Total References Indigenous Population (S) Disaggregated Population (D)	Time to treatment/Total delay (TTm/TD)	Time to diagnosis (TDx)	Patient delay (PD)	Health systems delay (HD)	Health system-treatment Delay (TmD)	Health system-diagnostic Delay (DCD)
		Total References: Read Across (S/D)					
All studies	Total references	6	5	15	4	3	4
	Total references assessing risk factors (S/D)	4 (1/3)	4 (3/1)	15 (7/8)	4 (2/2)	1 (0/1)	4 (1/3)
*Demographics*							
Increasing age/older age	Total references assessing risk factors (S/D)	3 (0/3)	1 (0/1)	10 (3/7)	3 (1/2)	1 (0/1)	2 (0/2)
	Delayed Event (S)			[36]^d^			
	Delayed Event (D)	[52]^c^	[28]^a,b^	[53]^c^	[40]^c^	[33]^b^	[33]^b^
Male	Total references assessing risk factors (S/D)	3 (0/3)	2 (1/1)	12 (4/8)	4 (2/2)	1 (0/1)	3 (0/3)
	Delayed Event (D)					[33]^b^	
	Timely Event (D)	[52]^b^		[53]^c^, [50]^b^, [38]^b,c^	[40]^b^		[39]^c^
Indigenous status	Total references assessing risk factors (S/D)	3 (0/3)	1 (0/1)	8 (0/8)	2 (0/2)	1 (0/1)	3 (0/3)
	Delayed Event (D)	[3]^c^, [52]^c^		[39]^b,c^, [3]^c^, [38]^c^, [54]^b^, [50]^b^		^b^ [33]	
	Timely Event (D)			[40]^b^	[40]^b^		
*Language, Education, and Culture*							
Language barrier	Total references assessing risk factors (S/D)	0 (0/0)	1 (1/0)	1 (0/1)	0 (0/0)	0 (0/0)	0 (0/0)
	Delayed Event (S)		[29, 50]^d^				
	Delayed Event (D)			[50]^d^			
Poor TB awareness and knowledge/illiteracy, misunderstanding about TB/low education status	Total references assessing risk factors (S/D)	3 (1/2)	1 (1/0)	10 (5/5)	3 (2/1)	0 (0/0)	1 (0/1)
	Delayed Event (S)	[41]^c^	[35]^c,d^	[37]^c^, [49]^d^, [41]^c^, [42]^b^	[41]^b,c^		
	Delayed Event (D)			[50]^d^, [3]^c^, [54]^b^			
*TB Specific*							
Cultural taboos, social isolation, and stigma	Total references assessing risk factors (S/D)	0 (0/0)	2 (2/0)	3 (2/1)	0 (0/0)	0 (0/0)	0 (0/0)
	Delayed Event (S)		[35]^c,d^, [31]^d^	[36]^d^			
	Delayed Event (D)			[50]^d^			
Smear-negative/Culture-positive/Have extra pulmonary TB	Total references assessing risk factors (S/D)	1 (0/1)	0 (0/0)	4 (1/3)	1 (0/1)	1 (0/1)	2 (1/1)
	Delayed Event (S)						[42]^b,c^
	Delayed Event (D)	[3]^b,c^			[3]^b,c^	[33]^b^	[33]^b^
	Timely Event (D)			[53]^b,c^			
Normal chest radiograph/not getting chest X-ray/misdiagnosis	Total references assessing risk factors (S/D)	1 (0/1)	1 (1/0)	0 (0/0)	0 (0/0)	1 (0/1)	2 (0/2)
	Delayed Event (S)		[35]^d^				
	Delayed Event (D)	[34]^c,d^				[33]^b^	[33]^b^, [38]^b^
*Health Care Access*							
Diagnosis at medical center/1st visit to health clinic/private clinics	Total references assessing risk factors (S/D)	3 (0/3)	1 (1/0)	4 (2/2)	2 (1/1)	1 (0/1)	2 (0/2)
	Delayed Event (S)			[41]^b,c^	[41]^b,c^		
	Delayed Event (D)	[3]^b,c^, [52]^b,c^		[51]^b^	[3]^b,c^		[33]^b^, [38]^b^
	Timely Event (D)					[33]^b^	
1st visit or general visit to traditional healer	Total references assessing risk factors (S/D)	3 (1/2)	1 (1/0)	6 (4/2)	1 (1/0)	0 (0/0)	0 (0/0)
	Delayed Event (S)	[41]^c^	[35]^c,d^	[36]^d^, [48]^b,c^, [41]^b,c^, [32]^d^	[37]^b^		
	Delayed Event (D)			[3]^b,c^, [54]^b^			
Self-treatment/not seeking medical care	Total references assessing risk factors (S/D)	1 (0/1)	1 (1/0)	5 (2/3)	2 (1/1)	0 (0/0)	0 (0/0)
	Delayed Event (S)		[29]^d^	[32]^d^			
	Delayed Event (D)			[3]^b,c^, [50]^d^, [54]^b^			
Distance to health facility/rural-remote	Total references assessing risk factors (S/D)	3 (0/3)	3 (2/1)	9 (5/4)	3 (2/1)	0 (0/0)	0 (0/0)
	Delayed Event (S)		[35]^d^, [31]^d^	[37]^c^, [36]^d^, [48]^b,c^, [41]^b,c^, [42]^b^	[41]^b,c^		
	Delayed Event (D)	[52]^b,c^, [34]^c,d^		[53]^c^, [3]^c^, [54]^b^, ^b,d^ [50]			
Transportation barrier (including Cost)	Total references assessing risk factors (S/D)	0 (0/0)	2 (2/0)	7 (4/3)	1 (1/0)	0 (0/0)	1 (0/1)
	Delayed Event (S)		[35]^d^, [29]^d^	[36]^d^, [48]^b,c^, [41]^b,c^			
	Delayed Event (D)			[39]^b,c^, [50]^d^, [51]^b,c^			
Cost of treatment/no health insurance/cost of hospital stay/loss of income	Total references assessing risk factors (S/D)	0 (0/0)	1 (1/0)	6 (4/2)	0 (0/0)	0 (0/0)	1 (0/1)
	Delayed Event (S)		[35]^d^	[37]^c^, [36]^d^, [41]^b,c^, [48]^c^			
	Delayed Event (D)			^b,c^ [39], [50]^d^			
Administrative delays i.e. verification for distribution of medicine, waiting for confirmation of diagnosis/referral for specialist, difficulty navigating health system	Total references assessing risk factors (S/D)	0 (0/0)	1 (1/0)	0 (0/0)	2 (1/1)	0 (0/0)	0 (0/0)
	Delayed Event (S)		[29]^d^		[37]^c^		
	Delayed Event (D)				[40]^d^		

^a^Time of onset to case notification, ^b^Multivariate, ^c^Descriptive/Univariate, ^d^Qualitative; References are not unique and can be in multiple columns

Age and gender had inconclusive associations across time to event measures (Table 5). In 63% (5/8) of studies, patient delays were significantly longer among Indigenous peoples compared to non-Indigenous persons. One study that described a shorter patient delay among Indigenous peoples highlighted some limitations in the interpretation of these results [40]. One study [42] that assessed patient delay in Ethiopia made comparisons within an Indigenous population group (not included in Table 5). This study described significantly longer delays among nomadic- compared to agro- pastoralists [42]. In 67% (2/3) of studies, total delay was greater among Indigenous peoples compared to non-Indigenous persons. Poor TB awareness, knowledge and/or education status was consistently associated with longer patient delay (7/10 studies; 70%). Cultural taboo, social isolation, and/or stigma was associated with longer patient (2/3 studies; 67%) and diagnosis (all 2 studies) delays. Type of health provider (i.e. traditional healer) first visited (all six studies) and self-treatment (4/5 studies; 80%) was consistently associated with longer patient delays. Distance to health facilities/remoteness (all 9 studies), transportation barriers (6/7 studies; 86%), and cost of treatment (all 6 studies) were associated with longer patient delays. Overall, risk factors assessed were generally evenly distributed between studies that solely focused on Indigenous and disaggregate populations. Type of health provider visited, transportation barriers, and cost of treatment represented risk factors that were assessed more in studies that focused on Indigenous compared to disaggregate populations.

## Discussion

There is an estimated population of at least 476 million Indigenous peoples across 90 countries worldwide [43]. While the incidence of TB is generally higher for Indigenous peoples, our search strategy resulted in only 24 studies (22 journal articles and 2 abstracts) that met the inclusion criteria for this systematic review. The estimated total (24–240 days), patient (20 days–2.5 years), and health system (3–34 days) delay for Indigenous peoples were within previously reported ranges that focused on PTB in low, middle, and high-income countries in the general population [10, 11, 44, 45]. One study estimating patient delay (2–3 years) that fell outside this range was conducted in the Solomon Islands, an extremely remote community with barriers to healthcare access due to distance, cost, and preference for traditional practices [36]. Over 60% of disaggregated studies described longer patient and/or total delay among Indigenous peoples compared to non-Indigenous persons despite shared socioeconomic challenges faced in medium (15–29 cases per 100,000 population) and high (> 30 cases per 100,000 population) TB incidence countries [45, 46].

Studies that estimated time to diagnosis and treatment of TB among Indigenous peoples were sparse in terms of volume and geographical coverage. Literature among Indigenous populations in regions such as Europe and South America were not represented in this systematic review. Half of the studies that met the inclusion criteria focused solely on Indigenous peoples [27, 29–32, 35–37, 41, 42, 47–49]. The remaining studies included these estimates stratified by population group [3, 28, 33, 34, 38–40, 44, 50–53]. The sparseness of literature highlights a need for more research to better understand time to diagnosis and treatment of TB among Indigenous peoples since their overall TB burden is generally higher than non-Indigenous persons [16].

Patient and/or total delay (10 studies) was longer among Indigenous peoples compared to non-Indigenous persons in 60% of disaggregated studies. Patient delay was strongly tied to total delay since it contributed the most to the overall pathway from symptom onset to treatment. One study [40] that described shorter patient delays among Indigenous Australians noted caution in the interpretation of results. Excluding this study would increase the previous result to 67% or two-thirds of disaggregate studies. The three studies [34, 51, 53] that showed no differences in patient and/or total delay between Indigenous peoples and non-Indigenous persons had samples mostly comprised of Indigenous peoples (> 85% of the total sample). These comparisons are impacted by unequal sample distributions and further strengthens observed differences in patient and/or total delay between Indigenous peoples and non-Indigenous persons.

Risk factors identified in this systematic review were consistent with those in other systematic reviews [4, 10, 44, 45]. Most studies examined risk factors associated with patient delay (63%; 15/24). Risk factors significantly associated with longer patient delays included having poor knowledge/awareness of TB, type of health provider first seen (traditional healer), self-treatment/not seeking care, distance to health facility, transportation challenges, and cost of treatment. These studies were distributed evenly across those that focused on aggregated and disaggregated studies. All disaggregated studies were conducted in countries with medium and high incidence of TB and 67% (4/6) of these showed longer patient delays between Indigenous peoples and non-Indigenous persons [3, 39, 50, 54]. In these countries, population differences associated with patient delay are blurred since they face similar circumstances of increased poverty, malnutrition, overcrowding, reduced access to healthcare, and poor education [45, 46]. In these circumstances, differences in risk factors associated with patient delay may not be easily differentiated due to shared socioeconomic challenges faced by Indigenous peoples and non-Indigenous persons. Since patient delay contributes the most towards the pathway from onset of symptoms to treatment, risk factor comparisons associated with other time to event measures between Indigenous peoples and non-Indigenous persons may not be similarly impacted by having shared socioeconomic circumstances alone. This highlights a literature gap that requires further assessment.

Although increasing knowledge and training of medical centre staff and other health professionals, increasing medical facilities, and subsidizing travel costs could improve factors related to healthcare access, socioeconomic barriers such as poverty, unemployment, poor housing, food insecurity, and transportation availability can impact the feasibility and acceptance of public health interventions [55]. Understanding the relationships between these barriers and public health interventions, specifically in countries or population groups that are most impacted is important to determining ways to improve healthcare access. For some Indigenous communities, traditional medicine represents an integral part of the culture and promoting the use of non-traditional medicines can be challenging [56, 57]. Collaborative knowledge sharing between traditional healers/practitioners and health professionals could help reduce patient delays in areas where this practice is more prevalent by encouraging people with symptoms related to TB to seek additional care from non-traditional medical practitioners.

Indigenous peoples in low TB incidence countries such as Canada, United States, Australia, and New Zealand have higher rates of poverty and chronic illness, and poor housing conditions and health care access [16, 19, 21, 28, 58–60], that similarly impact those in medium and high incidence countries. These comparisons were challenging to make since few studies were included from low incidence countries. Only two of these studies allowed for making comparisons between Indigenous and non-Indigenous [28, 40]. In these studies, time to event measures was estimated to be comparable between Indigenous and non-Indigenous groups. In the New Zealand study, 85% of the sample had a time to diagnosis estimate of over 4 weeks [28]. This percentile-based estimate and using 4-weeks as the cut-off value to make comparisons between population groups was difficult to interpret without knowing the overall distribution of estimates (e.g. mean/median). Recall of symptom onset was another bias identified in a study impacting Indigenous populations in Australia who experience higher case fatality rates indicative of advanced disease [40]. Overall comparisons and disparities of time to event measures between Indigenous and non-Indigenous populations in low incidence countries were limited and represents a gap in literature.

This systematic review has provided a general scope of the literature about time to diagnosis and treatment of TB among Indigenous peoples worldwide. The estimates obtained in this review provide a duration of time where appreciable interventions can be made to interrupt the transmission and reduce morbidity and/or mortality of TB. Although research methodologies and time to event definitions varied across studies, efforts were made to highlight appreciable differences and align definitions with terminology described in this review.

There were limitations in making comparisons of time to event measures of TB across studies due to scarcity of studies meeting inclusion criteria, inconsistent reporting of estimate types (mean, median, range, percentile type, and a pre-determined cut-off value), inclusion of uncertainty estimates, and variability of time to event definitions. The term delay was highly interpretable and often based on the data itself (e.g. median value) or arbitrarily selected, a priori. In this review, comparisons of time to event estimates between population groups were based on how authors chose to define a “delayed event” since additional data was limited. Cut-off values are useful for multivariate analysis, however, are subject to bias on how cut-off values are chosen. Reporting the spread (e.g. IQRs, standard deviations) and central tendency (e.g. mean/median) in future studies would allow for better comparisons between and within geographical regions and/or population groups since this type of data is often skewed and ‘delayed events’ of time to event measures are not yet standardized.

Similarly observed in previously conducted systematic reviews, the operationalization of time to event terminology was not consistent across literature [4, 10, 11, 13, 44]. Among studies assessed, 13% (3/24) [31, 32, 61] did not explicitly connect time to event terms to a definition and 33% (7/21) [28, 30, 33, 36, 38, 42, 53] used definitions that did not coincide with descriptions in Table 1. One study (written in mainly Mandarin) described patient delay using the term “treatment-delay time” [53]. These inconsistencies in defining time to event terminology highlight potential misclassification of terms if they were not well defined and missing studies during the literature search.

Half of studies (4/8) that described diagnosis as the end point (time to diagnosis and/or diagnostic delay) did not clearly defined the term ‘diagnosis’ [29, 35, 39, 42]. The definition of “diagnosis” date is complex since it can be made from bacteriological, histological, and/or clinical evidence [62]. In practice and in literature, “diagnosis” or date of diagnosis in one study may not be the same in another, which makes comparisons between studies challenging. The date of diagnosis is dependent on its definition and test(s) used, e.g. culture (two to eight weeks), nucleic acid amplification test (24–48 h), and based on history of exposure, suggestive symptoms, and/or a chest radiographic abnormality consistent with TB (clinical diagnosis) [62]. As such, time to diagnosis estimates could potentially be overestimated if diagnosis was defined as a positive culture, as opposed to a positive smear. Although uncommon, if there is a high level of suspicion and/or an infection control issue, it is possible that the date of treatment could occur prior to a positive diagnostic test result. In this case, it is unknown how the date of diagnosis would be reported. The use of treatment start date as the “diagnosis” date would better estimate the potential impact of on-going transmission and the development of new TB cases and allow for improved comparisons between studies.

Future studies describing TB trends and time to event measures would benefit from well-defined start and ends points, including central tendency (e.g. mean/median) and variance for estimates especially if a subjective a priori cut-off value is used, and avoiding where possible, diagnosis date as an endpoint and using treatment date instead. A need for standardization of what constitutes a ‘delayed event’ or the use of terminology that avoids ‘delay’ where possible can help prevent possible misinterpretations since this is fraught with subjective cut-off values. Examples of terminology would include time to treatment instead of total delay, respectively.

One third of studies in this systematic review included pulmonary TB in combination with extrapulmonary TB [3, 28–30, 32, 35, 36, 42]. In these studies, time to event estimates could be over-estimated [4] based on the sample distribution between those who have pulmonary and extrapulmonary TB. This review explicitly highlighted these differences in the forest plot (Fig. 2) to provide additional context for comparison across studies and types of time to event measures. This systematic review was limited to peer reviewed literature and/or abstracts (not including outbreak analyses). Indigenous cultures are diverse and can use different ways to transfer and share knowledge that are not always written. The impact of not including all possible forms of knowledge that describe time to event measures for TB among Indigenous populations is unknown. The systematic review included literature published up to 2019 and excludes potential biases related to access to health services during the COVID-19 pandemic (from 2020). Time to event measures are likely to have been impacted since a large drop in TB cases with an increase in TB deaths was observed globally in 2020 and 2021 [1]. Conducting a comparison of time to event measures among Indigenous peoples and non-Indigenous persons between literature prior and post-2019 may provide additional insights to previously identified and/or new risk factors since more advanced disease was observed from 2020.

## Conclusion

This is the first systematic review that aimed to describe time to diagnosis, treatment, and other time event measures of TB among Indigenous populations worldwide. While only 24 studies met the inclusion criteria, this systematic review has highlighted a need for more research especially into risk factors associated time to event measures among Indigenous peoples. Studies examining time to event estimates for TB would in general benefit from clear definitions of end points and the inclusion of variance and central tendency to allow for better comparisons between population groups and geographies. Time to event estimates among Indigenous peoples were generally within reported ranges based on previously conducted systematic review in the general population. However, among literature examined in this systematic review that stratified by Indigenous and non-Indigenous peoples, patient and total delay was longer compared to non-Indigenous persons in at least 60% of studies. Risk factors associated with patient delay were previously described in literature and shared between Indigenous peoples and non-Indigenous persons, especially in those conducted in medium and high incidence countries. In these countries, differences may be challenging to identify without more in-depth research since Indigenous and non-Indigenous population groups may face similar circumstances related to socioeconomic status and social determinants of health. The reduction of TB is multi-faceted and requires improved case finding, increased treatment completion rates, reducing risk factors associated with progression from latent infection to disease, and most importantly the prevention of new TB cases by interrupting transmission. Estimating time to diagnosis, treatment, and other time to event measures including addressing risk factors would have clear benefits to both Indigenous peoples and non-Indigenous persons in the prevention of TB.

## Supplementary Information

**Additional file 1: Appendix A1.** MeSH terms used by PubMed using themes outlined in Table 2.

## Data Availability

All data generated or analysed during this study are included in this published article.

## Abbreviations

DD: Diagnosis delay
DCD: Health system diagnostic delay
EPTB: Extrapulmonary tuberculosis
HD: Health system delay
IQR: Interquartile range
MeSH: Medical Subject Headings
PD: Patient delay
PTB: Pulmonary tuberculosis
TB: Tuberculosis
TD: Total delay
TDx: Time to diagnosis
TmD: Health system treatment delay
TTm: Time to treatment
