# Supplementary material for: Time to diagnosis and treatment of pulmonary tuberculosis in indigenous peoples: a systematic review
Source: BMC Infect Dis. 2023 Mar 7;23:131. doi: 10.1186/s12879-023-08098-y (PMC9989566; doi:10.1186/s12879-023-08098-y)
Supplement: Supplementary file 1 — Additional file 1: Appendix A1. MeSH terms used by PubMed using themes outlined in Table 2. [file 12879_2023_8098_MOESM1_ESM.docx]

**Appendix A1**. MeSH terms used by PubMed using themes outlined in Table 2

| Search Terms^[[1]](#footnote-1)^* |
| --- |
| (TB[All Fields] OR ("tuberculosis"[MeSH Terms] OR "tuberculosis"[All Fields]) OR ("tuberculosis, pulmonary"[MeSH Terms] OR ("tuberculosis"[All Fields] AND "pulmonary"[All Fields]) OR "pulmonary tuberculosis"[All Fields] OR ("pulmonary"[All Fields] AND "tb"[All Fields]) OR "pulmonary tb"[All Fields]) OR ("tuberculosis, pulmonary"[MeSH Terms] OR ("tuberculosis"[All Fields] AND "pulmonary"[All Fields]) OR "pulmonary tuberculosis"[All Fields] OR ("pulmonary"[All Fields] AND "tuberculosis"[All Fields]))) AND ("diagnosis delay"[All Fields] OR "diagnostic delay"[All Fields] OR "treatment delay"[All Fields] OR "time delay"[All Fields] OR "delay in diagnosis"[All Fields] OR "delay in treatment"[All Fields] OR "time to diagnosis"[All Fields] OR "time to treatment"[All Fields] OR "total delay"[All Fields] OR "patient delay"[All Fields] OR "health system delay"[All Fields] OR "health systems delay"[All Fields] OR "health provider delay"[All Fields] OR "doctor delay"[All Fields]) AND ((indigenous[All Fields] OR "indigenous people"[All Fields] OR "indigenous peoples"[All Fields] OR "indigenous population"[All Fields] OR "first nation"[All Fields] OR "first nations"[All Fields] OR ("inuits"[MeSH Terms] OR "inuits"[All Fields] OR "inuit"[All Fields]) OR metis[All Fields] OR metis[All Fields] OR aborigine[All Fields] OR aboriginal[All Fields] OR (torres[All Fields] AND strait[All Fields] AND islander[All Fields]) OR maori[All Fields] OR ("cook"[All Fields] AND islander[All Fields]) OR tribe[All Fields] OR tribal[All Fields] OR "eskimo"[All Fields] OR ("indians, north american"[MeSH Terms] OR ("indians"[All Fields] AND "north"[All Fields] AND "american"[All Fields]) OR "north american indians"[All Fields] OR ("american"[All Fields] AND "indian"[All Fields]) OR "american indian"[All Fields]) OR ("indians, north american"[MeSH Terms] OR ("indians"[All Fields] AND "north"[All Fields] AND "american"[All Fields]) OR "north american indians"[All Fields] OR ("native"[All Fields] AND "american"[All Fields]) OR "native american"[All Fields]) OR ("alaska natives"[MeSH Terms] OR ("alaska"[All Fields] AND "natives"[All Fields]) OR "alaska natives"[All Fields]) OR ("roma"[MeSH Terms] OR "roma"[All Fields]) OR bushmen[All Fields] OR herdsmen[All Fields] OR (hill[All Fields] AND ("persons"[MeSH Terms] OR "persons"[All Fields] OR "people"[All Fields])) OR ("amazona"[MeSH Terms] OR "amazona"[All Fields] OR "amazon"[All Fields]) OR lahu[All Fields] OR akha[All Fields] OR Mon[All Fields] OR Lua[All Fields] OR mbri[All Fields] OR karen[All Fields] OR hmong[All Fields] OR miao[All Fields] OR Hui[All Fields] OR (("minority groups"[MeSH Terms] OR ("minority"[All Fields] AND "groups"[All Fields]) OR "minority groups"[All Fields] OR "minority"[All Fields]) AND ("china"[MeSH Terms] OR "china"[All Fields])) OR Aka[All Fields] OR babongo[All Fields] OR bacwa[All Fields] OR bagyeli[All Fields] OR baka[All Fields] OR bakola[All Fields] OR bakoya[All Fields] OR bambuti[All Fields] OR batwa[All Fields] OR pygmy[All Fields] OR aasax[All Fields] OR akie[All Fields] OR aweer[All Fields] OR barabaig[All Fields] OR dahalo[All Fields] OR datoga[All Fields] OR elmolo[All Fields] OR hadzabe[All Fields] OR hadza[All Fields] OR maasai[All Fields] OR "ogiek"[All Fields] OR sandawe[All Fields] OR sengwer[All Fields] OR waata[All Fields] OR yaaku[All Fields] OR amazigh[All Fields] OR imazighn[All Fields] OR berbers[All Fields] OR tuareg[All Fields] OR afar[All Fields] OR Aka[All Fields] OR boranna[All Fields] OR dinka[All Fields] OR fulani[All Fields] OR kanuri[All Fields] OR karamajong[All Fields] OR manoj[All Fields] OR nuer[All Fields] OR peul[All Fields] OR pygmy[All Fields] OR tuareg[All Fields] OR tubu[All Fields] OR wodaabe[All Fields] OR "bassari"[All Fields] OR "bororo"[All Fields] OR daza[All Fields] OR "nemadi"[All Fields] OR "ogoni"[All Fields] OR teda[All Fields] OR khoekhoe[All Fields] OR khoikhoi[All Fields] OR "basarwa"[All Fields] OR khwe[All Fields] OR nama[All Fields] OR (San[All Fields] AND ("africa"[MeSH Terms] OR "africa"[All Fields])) OR tsumkwe[All Fields] OR ("inuits"[MeSH Terms] OR "inuits"[All Fields] OR "aleut"[All Fields]) OR "alutor"[All Fields] OR "chelkancy"[All Fields] OR chukchi[All Fields] OR "chulymcy"[All Fields] OR "dolgan"[All Fields] OR ency[All Fields] OR "evenk"[All Fields] OR "itelmen"[All Fields] OR kamchadal[All Fields] OR kereki[All Fields] OR kety[All Fields] OR khanty[All Fields] OR koryak[All Fields] OR kumandincy[All Fields] OR mansi[All Fields] OR "Nanaicy"[All Fields] OR "Negidalcy"[All Fields] OR nenets[All Fields] OR nganasan[All Fields] OR "nivkhy"[All Fields] OR "Orochi"[All Fields] OR "Oroki"[All Fields] OR "Saami"[All Fields] OR sami[All Fields] OR selkup[All Fields] OR "shorcy"[All Fields] OR "soioty"[All Fields] OR tazy[All Fields] OR "telengity"[All Fields] OR "teleuty"[All Fields] OR "tofolar"[All Fields] OR "tubolar"[All Fields] OR ("tuvin"[All Fields] AND "todjin"[All Fields]) OR "udege"[All Fields] OR "ulchi"[All Fields] OR veps[All Fields] OR cherokee[All Fields] OR navajo[All Fields] OR choctaw[All Fields] OR sioux[All Fields] OR chippewa[All Fields] OR (("greenland"[MeSH Terms] OR "greenland"[All Fields]) AND ("inuits"[MeSH Terms] OR "inuits"[All Fields] OR "inuit"[All Fields])) OR (siberian[All Fields] AND ("inuits"[MeSH Terms] OR "inuits"[All Fields] OR "inuit"[All Fields])) OR (wa's[All Fields] AND nyoongar[All Fields]) OR "wongi"[All Fields] OR koori[All Fields] OR "tangata whenua"[All Fields] OR adivasi[All Fields] OR himba[All Fields] OR hausa[All Fields] OR yoruba[All Fields] OR igbo[All Fields] OR pastoralist[All Fields] OR panara[All Fields] OR surui[All Fields] OR xavante[All Fields] OR "arawak"[All Fields] OR tsukano[All Fields] OR maku[All Fields] OR "arapaso"[All Fields] OR "baniwa"[All Fields] OR "desana"[All Fields] OR "kubeo"[All Fields] OR "hupda"[All Fields] OR tuyuca[All Fields] OR wakana[All Fields] OR "tariana"[All Fields] OR "tukano"[All Fields] OR "amazonic"[All Fields] OR shipibo[All Fields] OR matsiguenga[All Fields] OR aguaruna[All Fields] OR shawi[All Fields] OR huambisa[All Fields] OR yanesha[All Fields] OR (quichua[All Fields] AND amazonian[All Fields]) OR Achuar[All Fields] OR nomastshiguenga[All Fields] OR (quechua[All Fields] AND lamistas[All Fields]) OR amarakaire[All Fields] OR harakmbut[All Fields] OR shapra[All Fields] OR (aymara[All Fields] AND andes[All Fields]) OR (quechua[All Fields] AND andes[All Fields]) OR warao[All Fields] OR (("pain"[MeSH Terms] OR "pain"[All Fields] OR "ache"[All Fields]) AND ("population groups"[MeSH Terms] OR ("population"[All Fields] AND "groups"[All Fields]) OR "population groups"[All Fields] OR "natives"[All Fields])) OR (icana[All Fields] AND aiari[All Fields]) OR tiquie[All Fields] OR yanomami[All Fields] OR "rio negro"[All Fields] OR anu[All Fields] OR bari[All Fields] OR wayuu[All Fields] OR yukpa[All Fields] OR yanomami[All Fields] OR "trio indians"[All Fields] AND "bedouins"[All Fields]) OR (chukotka[All Fields] AND autonomous[All Fields] AND indigenous[All Fields]) OR (jawadhu[All Fields] AND hills[All Fields]) OR "scheduled tribes"[All Fields] OR "Forest people"[All Fields] OR (dongria[All Fields] AND kondh[All Fields]) OR (kutia[All Fields] AND kondh[All Fields]) OR (langia[All Fields] AND saora[All Fields]) OR (pandi[All Fields] AND bhuiyan[All Fields]) OR nicobarese[All Fields] OR gond[All Fields] OR (gond[All Fields] AND gawari[All Fields]) OR mana[All Fields] OR pawara[All Fields] OR "raj gond"[All Fields] OR bahria[All Fields] OR bhil[All Fields] OR baiga[All Fields] OR "pacific islanders"[All Fields] OR "e-lun chun"[All Fields] OR "papua new guineans"[All Fields] OR bai[All Fields] OR chaoxian[All Fields] OR kazakh[All Fields] OR Li[All Fields] OR manchu[All Fields] OR mongols[All Fields] OR menggu[All Fields] OR tujia[All Fields] OR uyghur[All Fields] OR zhuang[All Fields] OR dogon[All Fields] OR (brazillian[All Fields] AND native[All Fields] AND indian[All Fields]) OR chine[All Fields] OR (zulu[All Fields] AND state[All Fields] AND indigenous[All Fields]) OR (madhya[All Fields] AND pradesh[All Fields] AND tribal[All Fields]) OR "Native Hawaiian"[All Fields]) |

1. * Mesh Terms from PubMed Search for Tuberculosis AND Time to diagnosis or treatment AND Indigenous populations [↑](#footnote-ref-1)
